# Visual outcomes of posterior chamber intraocular lens intrascleral fixation in the setting of postoperative and posttraumatic aphakia

**DOI:** 10.1186/s12886-016-0228-y

**Published:** 2016-05-04

**Authors:** Dariusz Haszcz, Katarzyna Nowomiejska, Agnieszka Oleszczuk, Cesare Forlini, Matteo Forlini, Joanna Moneta-Wielgos, Ryszard Maciejewski, Katarzyna Michalska-Malecka, Anselm G. Jünemann, Robert Rejdak

**Affiliations:** Department of General Ophthalmology, Medical University, Lublin, Poland; Domus Nova Hospital, Ravenna, Italy; Institute of Ophthalmology, University of Parma, Parma, Italy; Human Anatomy Department, Medical University in Lublin, Lublin, Poland; Gibinski University Clinical Centre, Medical University of Silesia, Katowice, Poland; Department of Ophthalmology, University of Rostock, Rostock, Germany; Department of Experimental Pharmacology, Medical Research Centre, Polish Academy of Sciences, Warsaw, Poland

**Keywords:** Aphakia, Secondary intraocular lens implantation, Scleral fixation

## Abstract

**Background:**

Several techniques for fixation of the posterior chamber intraocular lens (IOL) have been developed. We evaluate long-term functional outcomes and safety of posterior chamber IOL implantation using Hoffman scleral haptic fixation and sutureless Sharioth technique in patients with posttraumatic and postoperative aphakia.

**Methods:**

This retrospective case-series included 42 eyes operated by one surgeon. The data including demographic data, ocular history, preoperative, early postoperative and final best corrected visual acuity (BCVA), rate of complications as well as postoperative IOL position were collected. The mean follow-up was 14.5 months. Hoffman haptic scleral fixation was performed in 31 eyes, Sharioth technique—in 11 eyes. Aphakia was due to eye trauma (19) or complicated cataract surgery (23).

**Results:**

Overall, the final BCVA improved in 26 eyes, did not change in 5 eyes, and worsened in 11 eyes. No significant differences in BCVA were found between groups operated with Hoffman scleral fixation and Sharioth technique. Postoperatively, we noticed two dislocations of IOL fixated using Sharioth technique and none after Hoffman technique. No severe complications were observed.

**Conclusion:**

Both transscleral fixation techniques are feasible methods of secondary IOL implantation in posttraumatic and postoperative aphakia. with low incidence of complications, however visual outcomes are diverse.

## Background

Surgical secondary artificial intraocular lens (IOL) implantation is a standard procedure both in posttraumatic and postoperative aphakia. The status of the posterior capsule may vary from intact to partially deficient or totally absent. Thus, the technique of implantation of IOL may vary from putting the lens into the bag [1] to suturing of IOL to iris or implantation to the anterior or posterior chamber [2–7]. If the anterior capsule is not damaged, the lens may be implanted to the sulcus [1]. Anterior chamber IOL carry high risk of postoperative complications as corneal endothelial damage, uveitis, glaucoma, hyphema (UGH) and cystoid macular edema [8, 9]. Suturing the IOL to the iris may result in iris chafing, uveitis, and pupillary constriction [10]. Furthermore, iris fixation is impossible in cases of significant iris trauma. Currently, if the posterior capsule is not present and if there is lack of iris tissue, most of IOLs are placed into posterior chamber and sutured to the sclera through the ciliary sulcus or pars plana.

The aim of this study was to estimate the visual outcomes and safety of two methods of secondary posterior chamber IOL implantation-a transscleral IOL haptic fixation using Hoffman technique or Sharioth scleral suturing technique-in patients with deficient posterior capsule support due to trauma or complicated cataract surgery.

## Methods

This retrospective study included patients, who had secondary IOL implantation surgery performed between March 2011 and December 2014 in the Department of General Ophthalmology in Lublin, Poland. The study was approved by the independent Ethics Committee at the Medical University in Lublin, Poland and performed in accordance with the Declaration of Helsinki.

This study included 42 eyes of 42 patients (15 women, 27 males). The mean age was 53.5 years ± 21.5 (SD) (range 13–85 years). The mean follow-up was 14.5 months ± 2.2 (SD) (range 12–16 months). Inclusion criteria were as follows: (1) total absence of capsular bag, (2) history of eye trauma or complicated cataract surgery causing aphakia, (3) regular 1 year follow-up. The preoperative diagnosis was as follows: post pars plana vitrectomy (PPV) due to intraocular foreign body (IOFB)-2 eyes, post PPV due to endophthalmitis-5 eyes, post blunt eye trauma-12 eyes and after complicated cataract surgery-23 eyes. Data collected included demographic data, ocular history, indication for surgery, preoperative and postoperative best corrected visual acuity (BCVA), intraocular pressure, detailed anterior and posterior segment evaluation using stereoscopic slit lamp biomicroscopy and indirect ophthalmoscopy. Patients were evaluated on the day 1, day 3, day 14, 3 months, and 12 months postoperatively. Intraocular lens position was assessed by a slit lamp examination with a dilated pupil, nonvisibility of the optic edge in a mid-dilated pupil of 4 mm was considered as a good centration. Final BCVA was the principal visual outcome indicator (expressed in Snellen decimal letters). It was reported as the percentage of eyes achieving BCVA of 0.5 or better, BCVA of 0.2–0.4, and BCVA of 0.1 or worse. Data were analyzed using *t*-test.

### Surgical techniques

All eyes were operated in local anesthesia (peribulbar injection of mixture of lignocaine, bupivacain and hylasis). Postoperative medication included topical drops (combined antibiotic and steroid) given 5 times daily and taped slowly for 4 weeks. Three kinds of IOL were implanted: Alcon MA60AC and Alcon MA60BM 3-piece, acrylic foldable IOL (Alcon International, United States) as well as Rayner 570C acrylic injectable, hydrophilic IOL (Rayner Intraocular Lenses, Ltd., Hove, East Sussex, United Kingdom).

Each procedure was performed by one surgeon (DH)- 31 patients underwent Hoffman technique and 11—Sharioth technique. All eyes underwent anterior vitrectomy during the primary surgery as a routine accompaniment. Additionally, a constant infusion was needed.

**Fig. 1:**
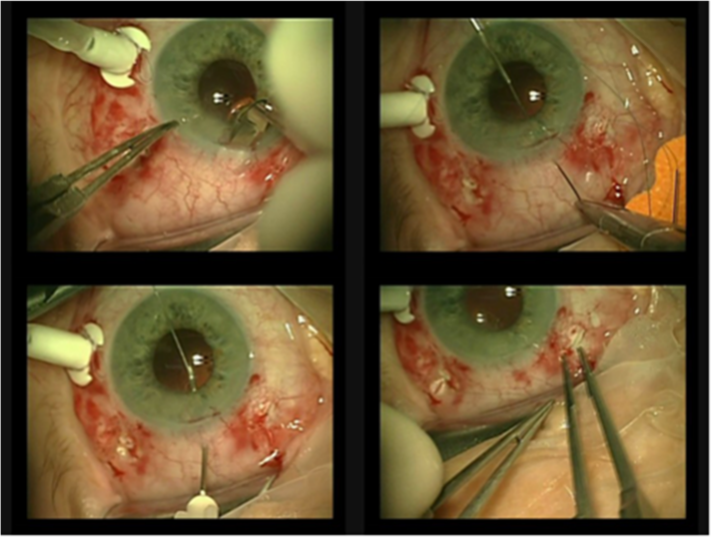
Hoffman technique-1) hollowing the tunnel in the sclera 2) a straight needle attached to a 10–0 polypropylene suture is passed through the conjuctiva and sclera in the pocket, posterior to the iris to the pupil, out through the opposite paracentes 3) the needle is passed back using hollow 26-G needle 4) after tying the haptic, the suture is tied into pocket

1. Hoffman technique (Fig. 1)

**Fig. 2:**
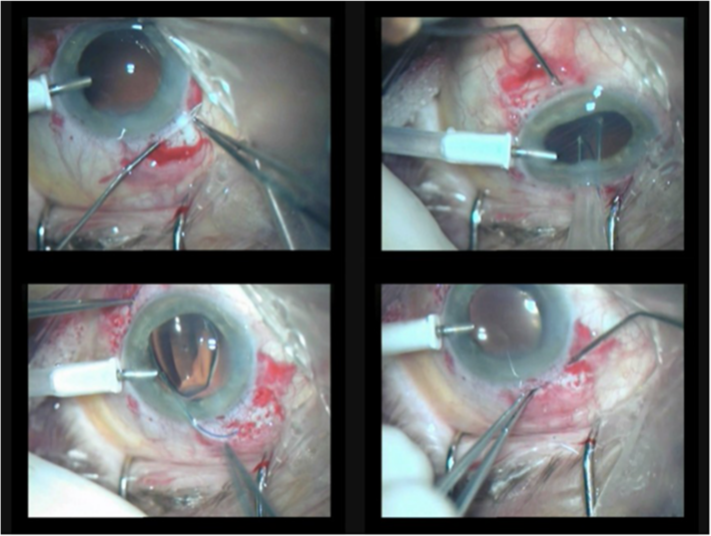
Scharioth technique-1) hollowing the intrascleral tunnel 2) inserting a 25-G needle at the end of the opposite intrascleral tunnel, insert the haptic of the PCIOL (posterior chamber intraocular lens) into the hollow needle (this maneuver can be performed using micropincet 25-G) 3) removal of the haptic out by withdrawing the needle, the other haptic is retrieved using tweezers 4) inserting the second haptic into opposite intrascleral tunnel via the barrel of the hollow needle

  First, a 3–4 mm wide corneal incision is made, 0.5 mm anterior to the limbus, at a depth of 0.3 mm. Using a crescent knife the tunnel is made 2–3 mm posterior to the limbus, creating the reverse sclera pocket. A straight needle attached to a 10–0 polypropylene suture is passed through the roof of the scleral pocket, posterior to the iris and to the pupil area, out through the opposite paracentesis. The needle is passed back in the barrel of a hollow 26-gauge needle. The same procedure is made on the opposite side. A corneal incision of 2.6 mm is performed for lens implantation. The loops of 10–0 polypropylene sutures are externalized through corneal incision and sutured to the haptics. Then the sutures outgoing through the sclera, are drawn out through the incisions (in scleral pockets) and tied. The knots are buried in sclera pockets and no conjunctiva suturing is needed [11].
2. Sharioth technique (Fig. 2)

- After peritomies are done, sclerectomy is made using a 25-gauge needle. Two 2.00–3.00 mm scleral tunnels are created parallel to the limbus 180° from each other. After the PCIOL (posterior chamber intraoocluar lens) is inserted, a 25-gauge needle is inserted at the end of one intrascleral tunnel, the haptic of the PCIOL is inserted into the hollow needle (this maneuver can be performed using micropincet 25-gauge).
- The haptic is removed out by withdrawing the needle and inserted into the scleral tunnel. The other haptic is retrieved using tweezers. This haptic is inserted into the opposite intrascleral tunnel. Finally, the haptics are suspended through the sclerectomies into these scleral tunnels and the peritomies are closed [12].

## Results

### Functional results

Overall, mean preoperative BCVA was 0.279 (range 0.025–1.0) whereas early postoperative BCVA was 0.249 (range 0.025–0.6) and late postoperative 0.354 (range 0.010–1.0) (*p* = 0.176) (Table 1). Mean preoperative BCVA was 0.291 (range 0.025–1.0) in the group operated with Hoffman technique and 0.245 (range 0.1–0.6) in the group managed with Sharioth technique. Mean early postoperative BCVA was 0.239 (range 0.025–0.600) and 0.279 (range 0.025–0.600), respectively (*p* = 0.557). Mean late postoperative BCVA was 0.297 (range 0.010–1.000) and 0.454 (range 0.010–0.900), respectively (*p* = 0.161).

**Table 1:** Demographic datao f patients and the functional results (BCVA - best-corrected visual acuity) of two techniques of posterior chamber intraocular lens implantation (Hoffman and Sharioth techniques)

	Hoffman scleral fixation (*n* = 31)	Sharioth technique (*n* = 11)
Mean age	52 years	55 years
Male/female ratio	22/9	6/5
Posttraumatic aphakia	16 patients	3 patients
Postoperative aphakia	15 patients	8 patients
Mean preoperative BCVA	0.291	0.245
Mean early postoperative BCVA	0.239	0.279
Mean final BCVA	0.297	0.454
Complications	None	2 dislocations

Overall, eighteen eyes (43 %) had a final BCVA of 0.5 or better, eighteen eyes (43 %) of 0.2 to 0.4, and six eyes (14 %) of worse than 0.1. Overall, the final BCVA improved in 26 eyes (62 %), did not change in 5 eyes (12 %), and worsened in 11 eyes (26 %). In the group of eyes with worsening of the visual acuity most of them (8 eyes) were posttraumatic.

### Complications

In the present study we noticed two dislocations of PCIOL fixated using Sharioth technique. First patient suffered from severe ocular trauma. After 2 months, dislocation of PCIOL was found. The Siepser knot was tied to the haptic and it was suspended through the scleral flap. Second patient had an ocular history of TPPV surgery after endophthalmitis. Originally, implant was fixated using Scharioth technique. After 1 month the PCIOL became luxated. Reoperation was performed using Siepser knot to tie the haptic to the scleral bed.

We have not observed any dislocation after Hoffman technique. Severe complications (eg. expulsive hemorrhage, retinal or choroidal detachment, prolonged inflammation, or secondary glaucoma) were not observed in our group of patients. No evidence of suture erosion was found.

## Discussion

In the present study we have shown the functional results and complication rate after two secondary PCIOL implantation methods in patients with posttraumatic and postoperative aphakia after 1-year follow-up. Many authors highlight the fact that transscleral fixation provides the most physiological placement of IOL in cases of absence of the lens capsule [5, 11, 12]. Sulcus placement without fixation to the sclera ensures early satisfactory outcomes, but significant complications (eg, erosion of the iris, UGH syndrome, iris pigment epithelial defects, decentration) may occur later [13, 14].

Angle-supported anterior chamber IOL have been rarely used due to numerous complications (endothelial cell loss, corneal decompensation, UGH). However, many of the problems were associated with the older closed loop anterior chamber IOLs and are not common in the newer open-loop single-piece anterior chamber IOLs [15].

Iris-claw IOLs may be a good alternative, however higher costs limit their extensive usage [16].

A large prospective study (176 patients) comparing different secondary IOL implantation techniques combined with penetrating keratoplasty showed that iris-suture fixation provided less complications (cystid macular oedema, glaucoma escalation, IOL dislocation, graft failure) than transscleral fixation [17].

Relatively new technique is fibrin-glue assisted sutureless fixation described by Argawal [18]. In this technique scleral flaps are made horizontally at 3 and 9 o’ clock (as also described for standard trans-scleral suture fixation procedures). One-year results showed good outcomes [19].

Several techniques for fixation of the posterior chamber IOL to the ciliary sulcus have been developed [2–5, 20]. Malbran and co-authors were the first to report transscleral sulcus fixation with sutures of posterior chamber lenses in aphakic patients who had had previous intracapsular cataract extraction [8]. Sharioth technique [12], performed in some of our patients, does not require any suturing. Two scleral tunnels created for suspension of the haptics of the PCIOL seem to be effective for transscleral fixation. This method avoids intraocular knots with free suture ends and minimises potential risk of iris chafing. Sharioth technique is technically more difficult than Hoffman technique, but allows to minimise intraocular manipulations. However, we noticed two cases of decentration of the PCIOL after 1 and 2 months (5 %) in eyes operated with Sharioth technique (one patient after severe ocular trauma and one after complicated retinal detachment surgery). Re-interventions were needed in both cases.

In our study most cases the PCIOL were stable without tilt. It is known that erosion of suture knots through the conjunctiva creates a communication between the extra- and intraocular environments, increasing the risk of contamination [2]. When knots are tied under the conjunctiva alone, the risk is up to 24 %, even scleral flaps are associated with the risk of 15 % [21].

Nottage and colleagues in a study of 69 patients after transscleral fixation observed glaucoma (5.8 %), cystic macular oedema (5.8 %), bullous keratopathy (4.3 %), retinal detachment (1.4 %), uveitis (1.4 %), keratitis (1.4 %) and choroidal haemorrhage (1.4 %) after 14 months of the follow-up [22]. They observed 1 suture erosion 2 years after surgery.

In the present series of 42 patients we observed deterioration of the visual acuity in one-fourth of cases. It is quite high percentage, although most of these cases were posttraumatic. However, 43 % of our patients had a final BCVA of 0.5 or better . Other studies describe better functional results. For example Kjeka and colleagues [23] report the mean preoperative BCVA 0.37, which improved to 0.5 postoperatively. At the end of follow-up, BCVA was unchanged or improved in 81 eyes (89.0 %), reduced by 2 Snellen lines in four eyes (4.4 %), and between finger counting and light perception in four eyes (4.4 %). The most serious complication was suprachoroidal haemorrhage, which occurred in two eyes, retinal detachment occurred in three eyes. In a study of Lanzetta [24] mean visual acuity was 0.29 preoperatively and 0.71 postoperatively after a mean follow-up of 14.2 months. A best corrected visual acuity of 0.5 or better was obtained in 12 eyes. In the recent study by Agrawal [25] the percentage of eyes with vision worsening after scleral fixation was 6.9 %. In our study we did not observe any severe complications such as corneal decompensation, cystoid macular edema, vitreous hemorrhage, retinal detachment, suprachoroidal hemorrhage, endophthalmitis, or glaucoma escalation.

## Conclusion

Both Hoffman and Sharioth techniques of posterior chamber IOL implantation are feasible methods of managing posttraumatic and postoperative aphakia. However, functional outcomes are diverse, especially in posttraumatic cases. Longer follow-up on a large population is required. Careful selection of patients and surgical method should be made before operation.

## Ethics approval and consent to participate

This study was approved by the Ethics Committee of the Medical University in Lublin, Poland. A written informed consent was obtained from all the participants after the study protocol had been explained.

## Consent to publish

A written informed consent to publish person’s data was sought along with the consent for participation into the study.

## Availability of data and materials

Data can be shared upon request.

## Abbreviations

BCVA: best corrected visual acuity
IOFB: intraocular foreign body
IOL: intraocular lens
PCIOL: posterior chamber intraocular lens
PPV: pars plana vitrectomy
